# Once more unto the leech: production of functional motor patterns in leech heart motor neurons

**DOI:** 10.1186/1471-2202-14-S1-P51

**Published:** 2013-07-08

**Authors:** Damon G Lamb, Ronald L Calabrese

**Affiliations:** 1 Department of Biology, Emory University, Atlanta, GA 30322, USA

## 

Leech heart motor neurons are the output component of the leech heartbeat pattern generator. Although the inhibitory synaptic input they receive from the central pattern generator (CPG) is the primary determinant of the rhythmic output they produce, the neurons themselves can contribute in a meaningful way to the patterns they produce [1-3]. In this study, we seek to understand the interaction of the inhibitory synaptic inputs, electrical coupling, and active membrane currents, as well as the interaction and balance between membrane currents. Towards this goal, we developed a 7 compartment GEneral NEural SImulation System (GENESIS 2.3) model which incorporated the currents known to be present in leech heart motor neurons. This model was parameterized by the maximal conductance for each of these currents, and an initial instance of this model was hand tuned to produce acceptable activity. We then used a multi-objective evolutionary algorithm (MOEA) to generate model instances which correctly produced a target output pattern within an acceptable error bound given the corresponding input pattern. The MOEA we used was previously applied to models of crustacean stomatogastric ganglion neurons [4]. We adapted it to run on a ~1000 node high-performance computing cluster (ELLIPSE, Emory IT) and to interact with our 7 compartment leech heart motor neuron model. This evolutionary algorithm independently selects the parents for each generation on each fitness metric separately, obviating the need to create a unified fitness function. Thus, a model instance that is excellent on only one metric can contribute to the subsequent generation even if it performs poorly on all other metrics. We executed 5 runs of this evolutionary algorithm with different, randomly selected initial populations and our post-hoc analyses examined the millions of resulting models from all runs of the MOEA. The fitness metrics we used to evaluate model instances are composed of two sets: output attributes that directly affect the muscles which the motor neurons innervate and attributes which capture other key identifying attributes of leech heart motor neurons. The former are the relative phase, as measured against the peristaltic interneuron in ganglion 4, and the intra-burst spike frequency for each neuron simulated. The latter are the duty cycle, spike height, and the slow wave height. The targets for each of these fitness metrics are drawn from our unique database of complete, individual-animal sets of spike time pattern, synaptic weight profile, and output pattern for leech heart motor neurons in ganglia 8 and 12. We have 431 model instances which met our targets on all fitness metrics and thousands that achieved the targets for only ganglia 8 or 12, but not both. We examined the contribution and balance of currents in these models and have found relationships between sets of currents and a strong differential effect of electrical coupling between pairs of leech heart motor neurons in different ganglia.

## References

[B1] Garcia PS Wright TM Cunningham IR Calabrese RL Using a model to assess the role of the spatiotemporal pattern of inhibitory input and intrasegmental electrical coupling in the intersegmental and side-to-side coordination of motor neurons by the leech heartbeat central pattern generator J Neurophysiol 2008 100 3 1354 1371 10.1152/jn.90579.2008 18579654 PMC2544471

[B2] Wright TM Calabrese RL Contribution of motoneuron intrinsic properties to fictive motor pattern generation J Neurophysiol 2011 106 2 538 553 10.1152/jn.00101.2011 21562194 PMC3154826

[B3] Wright TM Calabrese RL Patterns of presynaptic activity and synaptic strength interact to produce motor output J Neurosci 2011 31 48 17555 17571 10.1523/JNEUROSCI.4723-11.2011 22131417 PMC4127079

[B4] Smolinski T Boratyn G Milanova M Buchanan R Prinz A Rajapakse J, Wong L, Acharya R Hybridization of Independent Component Analysis, Rough Sets, and Multi-Objective Evolutionary Algorithms for Classificatory Decomposition of Cortical Evoked Potentials Pattern Recognition in Bioinformatics Volume 4146 2006 Springer Berlin Heidelberg 174 183

